# 9-Phenyl-10*H*-acridinium trifluoro­methane­sulfonate

**DOI:** 10.1107/S1600536810040900

**Published:** 2010-10-20

**Authors:** Damian Trzybiński, Beata Zadykowicz, Karol Krzymiński, Artur Sikorski, Jerzy Błażejowski

**Affiliations:** a Faculty of Chemistry, University of Gdańsk, J. Sobieskiego 18, 80-952 Gdańsk, Poland

## Abstract

In the crystal structure of the title compound, C_19_H_14_N^+^·CF_3_SO_3_^−^, the cations are linked to each other by very weak C—H⋯π inter­actions, while the cations and anions are connected by N—H⋯O, C—H⋯O and S—O⋯π inter­actions. The acridine ring system and the phenyl ring are oriented at an angle of 80.1 (1)° with respect to each other. The mean planes of adjacent acridine units are either parallel or inclined at an angle of 35.6 (1)°. The trifluoro­methane­sulfonate anions are disordered over two positions; the site occupancy factors are 0.591 (8) and 0.409 (8).

## Related literature

For general background to chemiluminescence, see: Sato (1996 ▶); Wróblewska *et al.* (2004 ▶); Zomer & Jacquemijns (2001 ▶). For related structures, see: Huta *et al.* (2002 ▶); Magnussen *et al.* (2007 ▶); Stowell *et al.* (1991 ▶); Toma *et al.* (1994 ▶); Trzybiński *et al.* (2010 ▶); Zadykowicz *et al.* (2009*a* ▶,*b* ▶). For inter­molecular inter­actions, see: Aakeröy *et al.* (1992 ▶); Dorn *et al.* (2005 ▶); Novoa *et al.* (2006 ▶); Takahashi *et al.* (2001 ▶). For the synthesis, see: Tsuge *et al.* (1965 ▶); Zadykowicz *et al.* (2009*b* ▶). For the treatment of the disorder, see: Müller *et al.* (2006 ▶).
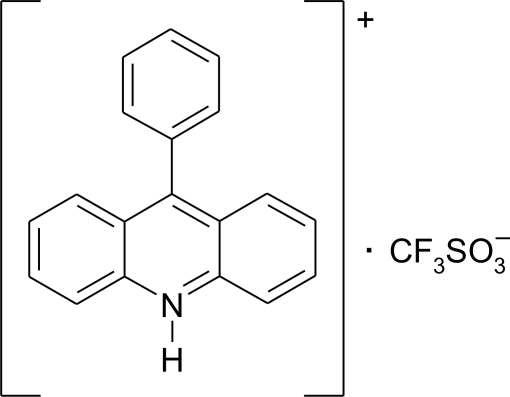


## Experimental

### 

#### Crystal data

C_19_H_14_N^+^·CF_3_SO_3_^−^
*M* *_r_* = 405.39
Monoclinic, 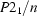

*a* = 9.7064 (5) Å
*b* = 8.9220 (3) Å
*c* = 21.8665 (9) Å
β = 100.902 (4)°
*V* = 1859.47 (14) Å^3^
*Z* = 4
Mo *K*α radiation
μ = 0.22 mm^−1^
*T* = 295 K
0.40 × 0.15 × 0.04 mm

#### Data collection

Oxford Diffraction Gemini R Ultra Ruby CCD diffractometer
Absorption correction: multi-scan (*CrysAlis RED*; Oxford Diffraction, 2008 ▶) *T*_min_ = 0.895, *T*_max_ = 1.000
35783 measured reflections
3296 independent reflections
1565 reflections with *I* > 2σ(*I*)
*R*_int_ = 0.066

#### Refinement

*R*[*F* ^2^ > 2σ(*F* ^2^)] = 0.059
*wR*(*F* ^2^) = 0.184
*S* = 1.03
3296 reflections
281 parameters
18 restraints
H atoms treated by a mixture of independent and constrained refinement
Δρ_max_ = 0.31 e Å^−3^
Δρ_min_ = −0.30 e Å^−3^

### 

Data collection: *CrysAlis CCD* (Oxford Diffraction, 2008 ▶); cell refinement: *CrysAlis RED* (Oxford Diffraction, 2008 ▶); data reduction: *CrysAlis RED*; program(s) used to solve structure: *SHELXS97* (Sheldrick, 2008 ▶); program(s) used to refine structure: *SHELXL97* (Sheldrick, 2008 ▶); molecular graphics: *ORTEP-3* (Farrugia, 1997 ▶); software used to prepare material for publication: *SHELXL97* and *PLATON* (Spek, 2009 ▶).

## Supplementary Material

Crystal structure: contains datablocks global, I. DOI: 10.1107/S1600536810040900/vm2046sup1.cif

Structure factors: contains datablocks I. DOI: 10.1107/S1600536810040900/vm2046Isup2.hkl

Additional supplementary materials:  crystallographic information; 3D view; checkCIF report

## Figures and Tables

**Table 1:** Hydrogen-bond geometry (Å, °) *Cg*2 is the centroid of the C1–C4/C11/C12 ring.

*D*—H⋯*A*	*D*—H	H⋯*A*	*D*⋯*A*	*D*—H⋯*A*
C3—H3⋯O24*A*^i^	0.93	2.44	3.333 (9)	160
C4—H4⋯O22*A*	0.93	2.59	3.348 (8)	139
C5—H5⋯O23*A*	0.93	2.28	3.154 (9)	157
N10—H10⋯O22*A*	0.83 (4)	2.43 (4)	3.198 (9)	154 (3)
C17—H17⋯*Cg*2^ii^	0.93	2.99	3.632 (7)	127
C20—H20⋯O24*A*^iii^	0.93	2.56	3.461 (9)	162

Symmetry codes: (i) 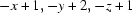; (ii) 
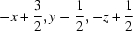
; (iii) 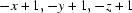.

**Table 2:** S–O⋯π inter­actions (Å, °) *Cg*1 and *Cg*3 are the centroids of the C9/N10/C11–C14 and C5–C8/C13/C14 rings, respectively.

*X*	*I*	*J*	*I*⋯*J*	*X*⋯*J*	*X*–*I*⋯*J*
S21	O23*A*	*Cg*1^iii^	3.125 (11)	3.923 (2)	114.7 (5)
S21	O22*B*	*Cg*3^iii^	3.387 (7)	3.990 (2)	104.7 (3)
S21	O23*B*	*Cg*1^iii^	3.159 (9)	3.923 (2)	111.8 (4)

Symmetry code: (iii) 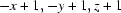.

**Fig. 1.**
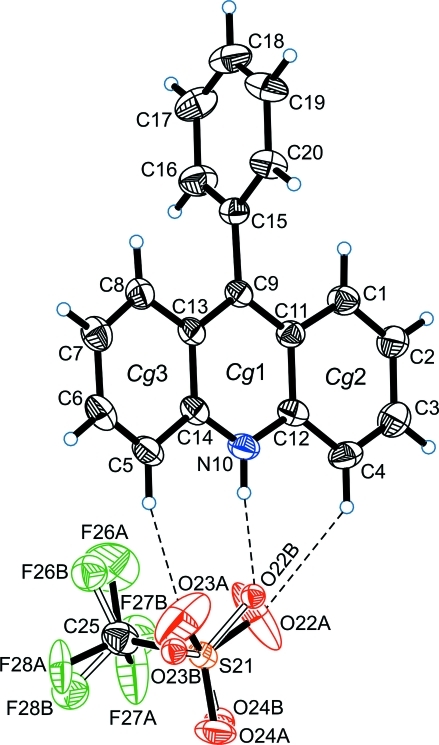
The molecular structure of the title compound showing the atom-labeling scheme. The N–H···O and C–H···O interactions are represented by dashed lines. Displacement ellipsoids are drawn at the 25% probability level and H atoms are shown as small spheres of arbitrary radius. Cg1, Cg2 and Cg3 denote the ring centroids.

**Fig. 2.**
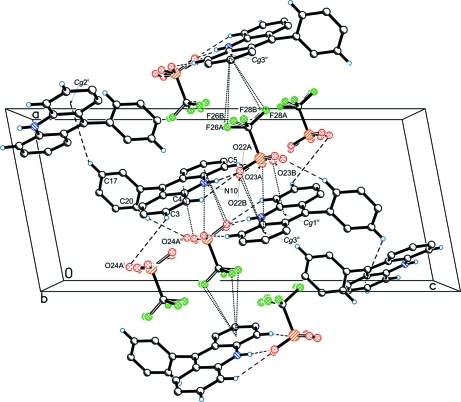
The arrangement of the ions in the crystal structure. The N–H···O, C–H···O and C–H···π interactions are represented by dashed lines, the C–F···π and S–O···π contacts by dotted lines. H atoms not involved in interactions have been omitted. Cg1, Cg2 and Cg3 denote the ring centroids. [Symmetry codes: (i) –x + 1, –y + 2, –z + 1; (ii) –x + 3/2, y – 1/2, –z + 1/2; (iii) –x + 1, –y + 1, –z + 1; (iv) –x + 2, –y + 1, –z + 1.]

## supplementary crystallographic information

### Comment

Acridinium cations containing various substituents at position 9 and alkyl-substituted at the endocyclic *N* atom undergo oxidation in alkaline media, resulting in electronically excited *N*-alkyl-9-acridinones. Light emission by these species is the basis of chemiluminesce (Zomer & Jacquemijns, 2001; Wróblewska *et al.*, 2004) which is influenced principally by the substituent at position 9. In the search for derivatives with enhanced chemiluminescence we investigated compounds in which C9 is substituted by substituents other than phenoxycarbonyl, which we have already investigated extensively (Huta *et al.*, 2002; Zadykowicz *et al.*, 2009*a*,*b*; Trzybiński *et al.*, 2010).

The compound whose crystal structure is reported here – 9-phenyl-10*H*-acridinium trifluoromethanesulfonate – was obtained by the reaction of 9-phenylacridine with methyl trifluoromethanesulfonate, which usually leads to the quaternarization of the endocyclic *N* atom (Sato, 1996). Since protonation at the endocyclic *N* atom took place, we presume that traces of water caused the conversion of methyl trifluoromethanesulfonate to trifluoromethanesulfonic acid and methanol, and the reaction of the former entity with 9-phenylacridine. The cations of the title compound have a protonated endocyclic *N* atom, which enable their reaction with oxidants. It is worth mentioning that salts containing protonated 9-phenylacridines exhibit interesting chromoisomeric features and potential chemiluminogenic ability (Toma *et al.*, 1994).

In the cation of the title compound (Fig. 1), bond lengths and angles are similar to the ones found in 9-phenyl-10*H*-acridinium chloride (Stowell *et al.*, 1991) and sulfate (Toma *et al.*, 1994), and are typical of other acridine-based derivatives (Trzybiński *et al.*, 2010). With respective average deviations from planarity of 0.0404 (3) Å and 0.0015 (3) Å, the acridine and benzene rings are oriented at 80.0 (1)° (65 (3)° in 9-phenyl-10*H*-acridinium chloride (Stowell *et al.*, 1991) and 62.5 (1)° or 62.6 (1)° in 9-phenyl-10*H*-acridinium sulfate (Toma *et al.*, 1994)). The mean planes of adjacent acridine moieties are either parallel (remain at an angle of 0.0 (1)°) or inclined at an angle of 35.6 (1)°. The trifluoromethanesulfonate anions are disordered over two positions with site occupancy factors of 0.591 (8) and 0.409 (8) [similar disorder was found in pentaaquaoxovanadium(IV)bis(trifluoromethanesulfonate) (Magnussen *et al.*, 2007)].

In the crystal structure, cations are linked by C–H···π interactions (Table 1, Fig. 2) and cations and anions by N–H···O, C–H···O (Table 1, Figs. 1 and 2), C–F···π and S–O···π (Table 2, Fig. 2) interactions. N–H···O (Aakeröy *et al.*, 1992) and C–H···O (Novoa *et al.* 2006) interactions are of the hydrogen bond type. The C–H···π interactions should be of an attractive nature (Takahashi *et al.*, 2001), like the C–F···π (Dorn *et al.*, 2005) and S–O···π (Dorn *et al.*, 2005) interactions. The crystal structure is stabilized by a network of these short-range specific interactions and by long-range electrostatic interactions between the ions.

### Experimental

9-Phenylacridine was synthesized by heating a mixture of *N*-phenylaniline with an equimolar amount of benzoic acid, both dispersed in molten zinc chloride (493 K, 26 h) (Tsuge *et al.*, 1965). The crude product was purified by gravitational chromatography (SiO_2_, n-hexane-ethyl acetate, 5:1 *v*/*v*). 9-Phenyl-10*H*-acridinium trifluoromethanesulfonate was obtained by dissolving 9-phenylacridine and methyl trifluoromethanesulfonate (fivefold molar excess) in anhydrous dichloromethane and leaving the mixture for 3 h (Ar atmosphere, room temperature) (Zadykowicz *et al.*, 2009*b*). The crude salt was dissolved in a small amount of ethanol, filtered, and precipitated with a 25 *v*/*v* excess of diethyl ether. Yellow crystals suitable for X-ray analysis were grown from absolute ethanol solution (m.p. 429–431 K).

### Refinement

The H atom at N10 was refined freely with *U*_iso_(H) = 1.2*U*_eq_(N10). Other H atoms were positioned geometrically, with C—H = 0.93 Å and constrained to ride on their parent atoms with *U*_iso_(H) = 1.2*U*_eq_(C). The trifluoromethanesulfonate anions were found to be disordered. The structure was resolved on the assumption that the C25–S21 bond is a common one and that the SO_3_ and CF_3_ groups occupy two positions – A and B. The occupancy ratio was initially determined by isotropic refinement of the disordered site and the structure was refined freely during the subsequent anisotropic refinement of A. The disordered SO_3_ and CF_3_ groups were refined assuming two ideal triangles for A and B, respectively, with a restrained standard deviation of 0.001 Å for the O···O and F···F distances (SADI instruction in *SHELXL97*) (Müller *et al.*, 2006).

### Crystal data

**Table:**

C_19_H_14_N^+^·CF_3_SO_3_^−^	*F*(000) = 832
*M**_r_* = 405.39	*D*_x_ = 1.448 Mg m^−^^3^
Monoclinic, *P*2_1_/*n*	Mo *K*α radiation, λ = 0.71073 Å
Hall symbol: -P 2yn	Cell parameters from 7503 reflections
*a* = 9.7064 (5) Å	θ = 3.0–29.2°
*b* = 8.9220 (3) Å	µ = 0.22 mm^−^^1^
*c* = 21.8665 (9) Å	*T* = 295 K
β = 100.902 (4)°	Plate, yellow
*V* = 1859.47 (14) Å^3^	0.40 × 0.15 × 0.04 mm
*Z* = 4	

### Data collection

**Table:**

Oxford Diffraction Gemini R Ultra Ruby CCD diffractometer	3296 independent reflections
Radiation source: Enhanced (Mo) X-ray Source	1565 reflections with *I* > 2σ(*I*)
graphite	*R*_int_ = 0.066
Detector resolution: 10.4002 pixels mm^-1^	θ_max_ = 25.1°, θ_min_ = 3.0°
ω scans	*h* = −11→11
Absorption correction: multi-scan (*CrysAlis RED*; Oxford Diffraction, 2008)	*k* = −10→10
*T*_min_ = 0.895, *T*_max_ = 1.000	*l* = −26→26
35783 measured reflections	

### Refinement

**Table:**

Refinement on *F*^2^	Primary atom site location: structure-invariant direct methods
Least-squares matrix: full	Secondary atom site location: difference Fourier map
*R*[*F*^2^ > 2σ(*F*^2^)] = 0.059	Hydrogen site location: inferred from neighbouring sites
*wR*(*F*^2^) = 0.184	H atoms treated by a mixture of independent and constrained refinement
*S* = 1.03	*w* = 1/[σ^2^(*F*_o_^2^) + (0.098*P*)^2^] where *P* = (*F*_o_^2^ + 2*F*_c_^2^)/3
3296 reflections	(Δ/σ)_max_ = 0.001
281 parameters	Δρ_max_ = 0.31 e Å^−^^3^
18 restraints	Δρ_min_ = −0.30 e Å^−^^3^

### Special details

**Table:**

Geometry. All esds (except the esd in the dihedral angle between two l.s. planes) are estimated using the full covariance matrix. The cell esds are taken into account individually in the estimation of esds in distances, angles and torsion angles; correlations between esds in cell parameters are only used when they are defined by crystal symmetry. An approximate (isotropic) treatment of cell esds is used for estimating esds involving l.s. planes.

### Fractional atomic coordinates and isotropic or equivalent isotropic displacement parameters (Å^2^)

**Table:**

	*x*	*y*	*z*	*U*_iso_*/*U*_eq_	Occ. (<1)
C1	0.4643 (5)	0.6284 (4)	0.27061 (19)	0.0756 (12)	
H1	0.4534	0.5817	0.2320	0.091*	
C2	0.4201 (5)	0.7706 (4)	0.2742 (2)	0.0851 (13)	
H2	0.3797	0.8207	0.2379	0.102*	
C3	0.4339 (5)	0.8442 (5)	0.3312 (3)	0.0909 (14)	
H3	0.4027	0.9425	0.3325	0.109*	
C4	0.4925 (5)	0.7740 (4)	0.3849 (2)	0.0791 (12)	
H4	0.5011	0.8233	0.4229	0.095*	
C5	0.6975 (5)	0.3417 (5)	0.4947 (2)	0.0798 (12)	
H5	0.7091	0.3970	0.5314	0.096*	
C6	0.7339 (5)	0.1953 (6)	0.4955 (2)	0.0906 (14)	
H6	0.7685	0.1494	0.5335	0.109*	
C7	0.7211 (5)	0.1098 (5)	0.4403 (2)	0.0840 (13)	
H7	0.7474	0.0094	0.4426	0.101*	
C8	0.6709 (4)	0.1728 (4)	0.3841 (2)	0.0713 (11)	
H8	0.6639	0.1162	0.3479	0.086*	
C9	0.5736 (4)	0.3985 (4)	0.32456 (16)	0.0580 (10)	
N10	0.6000 (4)	0.5519 (4)	0.43513 (16)	0.0716 (10)	
H10	0.611 (5)	0.600 (4)	0.4680 (19)	0.086*	
C11	0.5279 (4)	0.5477 (4)	0.32528 (17)	0.0615 (10)	
C12	0.5403 (4)	0.6246 (4)	0.38231 (18)	0.0661 (11)	
C13	0.6286 (4)	0.3275 (4)	0.38061 (18)	0.0608 (10)	
C14	0.6417 (4)	0.4080 (4)	0.43728 (18)	0.0660 (11)	
C15	0.5655 (4)	0.3198 (4)	0.26420 (17)	0.0625 (10)	
C16	0.6630 (6)	0.3472 (5)	0.2280 (2)	0.0960 (15)	
H16	0.7368	0.4127	0.2420	0.115*	
C17	0.6528 (7)	0.2784 (6)	0.1707 (3)	0.1125 (18)	
H17	0.7200	0.2972	0.1465	0.135*	
C18	0.5456 (8)	0.1840 (6)	0.1498 (2)	0.1074 (18)	
H18	0.5393	0.1384	0.1111	0.129*	
C19	0.4483 (7)	0.1554 (5)	0.1842 (2)	0.1045 (17)	
H19	0.3748	0.0901	0.1696	0.125*	
C20	0.4578 (5)	0.2241 (4)	0.24210 (19)	0.0828 (13)	
H20	0.3900	0.2045	0.2660	0.099*	
S21	0.73545 (11)	0.75830 (11)	0.58719 (4)	0.0664 (4)	
O22A	0.6636 (9)	0.8243 (11)	0.5314 (3)	0.218 (6)	0.591 (8)
O22B	0.6445 (7)	0.7598 (6)	0.5283 (3)	0.0458 (19)*	0.409 (8)
O23A	0.7106 (11)	0.6045 (7)	0.5921 (4)	0.218 (6)	0.591 (8)
O23B	0.7280 (9)	0.6199 (7)	0.6195 (2)	0.052 (2)*	0.409 (8)
O24A	0.7300 (9)	0.8420 (9)	0.6409 (3)	0.136 (3)	0.591 (8)
O24B	0.7450 (11)	0.8840 (7)	0.6215 (3)	0.074 (2)*	0.409 (8)
C25	0.9128 (6)	0.7584 (6)	0.5725 (3)	0.0964 (15)	
F26A	0.9168 (11)	0.6857 (11)	0.5215 (3)	0.298 (9)	0.591 (8)
F27A	0.9485 (9)	0.8946 (5)	0.5642 (4)	0.200 (5)	0.591 (8)
F28A	1.0032 (6)	0.7006 (8)	0.6159 (3)	0.126 (3)	0.591 (8)
F26B	0.9370 (13)	0.6408 (7)	0.5392 (4)	0.102 (3)*	0.409 (8)
F27B	0.940 (2)	0.8798 (10)	0.5386 (3)	0.134 (4)*	0.409 (8)
F28B	1.0068 (14)	0.7612 (8)	0.6237 (4)	0.108 (4)*	0.409 (8)

### Atomic displacement parameters (Å^2^)

**Table:**

	*U*^11^	*U*^22^	*U*^33^	*U*^12^	*U*^13^	*U*^23^
C1	0.089 (3)	0.070 (2)	0.066 (3)	0.001 (2)	0.010 (2)	0.001 (2)
C2	0.099 (3)	0.069 (3)	0.083 (3)	0.015 (2)	0.007 (3)	0.002 (2)
C3	0.098 (4)	0.071 (3)	0.105 (4)	0.009 (2)	0.022 (3)	0.003 (3)
C4	0.088 (3)	0.069 (3)	0.084 (3)	−0.008 (2)	0.023 (3)	−0.024 (2)
C5	0.073 (3)	0.098 (3)	0.061 (3)	−0.005 (2)	−0.004 (2)	−0.004 (2)
C6	0.076 (3)	0.116 (4)	0.071 (3)	−0.003 (3)	−0.008 (2)	0.017 (3)
C7	0.080 (3)	0.078 (3)	0.088 (3)	−0.002 (2)	0.002 (3)	0.012 (3)
C8	0.067 (3)	0.069 (2)	0.074 (3)	0.001 (2)	0.003 (2)	0.004 (2)
C9	0.056 (2)	0.061 (2)	0.056 (2)	−0.0061 (17)	0.0074 (19)	0.0001 (18)
N10	0.071 (2)	0.078 (2)	0.063 (2)	−0.0116 (17)	0.0054 (19)	−0.0177 (18)
C11	0.061 (2)	0.060 (2)	0.064 (2)	−0.0055 (17)	0.0114 (19)	−0.0064 (19)
C12	0.069 (3)	0.068 (2)	0.059 (3)	−0.0110 (19)	0.007 (2)	−0.006 (2)
C13	0.053 (2)	0.067 (2)	0.060 (3)	−0.0068 (17)	0.0047 (19)	−0.001 (2)
C14	0.057 (3)	0.074 (3)	0.063 (3)	−0.0089 (19)	0.002 (2)	−0.001 (2)
C15	0.073 (3)	0.057 (2)	0.057 (2)	−0.0023 (19)	0.011 (2)	−0.0020 (18)
C16	0.100 (4)	0.106 (3)	0.088 (3)	−0.018 (3)	0.034 (3)	−0.020 (3)
C17	0.136 (5)	0.112 (4)	0.105 (4)	−0.001 (4)	0.064 (4)	−0.018 (3)
C18	0.166 (6)	0.089 (3)	0.072 (3)	0.014 (4)	0.034 (4)	−0.010 (3)
C19	0.140 (5)	0.092 (3)	0.072 (3)	−0.029 (3)	−0.004 (3)	−0.015 (3)
C20	0.098 (3)	0.088 (3)	0.063 (3)	−0.021 (2)	0.015 (2)	−0.007 (2)
S21	0.0669 (7)	0.0723 (7)	0.0562 (6)	−0.0034 (5)	0.0017 (5)	−0.0060 (5)
O22A	0.114 (6)	0.346 (13)	0.158 (7)	−0.095 (8)	−0.068 (5)	0.157 (8)
O23A	0.170 (9)	0.106 (5)	0.417 (17)	−0.067 (5)	0.158 (11)	−0.110 (8)
O24A	0.105 (5)	0.222 (8)	0.079 (5)	−0.008 (6)	0.011 (4)	−0.081 (5)
C25	0.089 (4)	0.111 (4)	0.089 (4)	−0.023 (3)	0.016 (3)	−0.006 (3)
F26A	0.155 (9)	0.60 (3)	0.167 (8)	−0.020 (12)	0.108 (8)	−0.114 (11)
F27A	0.068 (4)	0.171 (6)	0.355 (12)	−0.011 (4)	0.028 (7)	0.181 (8)
F28A	0.056 (3)	0.101 (4)	0.209 (7)	0.025 (3)	−0.002 (3)	0.055 (4)

### Geometric parameters (Å, °)

**Table:**

C1—C2	1.347 (5)	C13—C14	1.417 (5)
C1—C11	1.431 (5)	C15—C20	1.365 (5)
C1—H1	0.9300	C15—C16	1.367 (6)
C2—C3	1.393 (6)	C16—C17	1.381 (6)
C2—H2	0.9300	C16—H16	0.9300
C3—C4	1.358 (6)	C17—C18	1.349 (8)
C3—H3	0.9300	C17—H17	0.9300
C4—C12	1.415 (5)	C18—C19	1.339 (7)
C4—H4	0.9300	C18—H18	0.9300
C5—C6	1.353 (6)	C19—C20	1.394 (6)
C5—C14	1.401 (5)	C19—H19	0.9300
C5—H5	0.9300	C20—H20	0.9300
C6—C7	1.412 (6)	S21—O24B	1.342 (6)
C6—H6	0.9300	S21—O23A	1.401 (7)
C7—C8	1.356 (5)	S21—O24A	1.401 (5)
C7—H7	0.9300	S21—O22A	1.413 (6)
C8—C13	1.438 (5)	S21—O22B	1.417 (6)
C8—H8	0.9300	S21—O23B	1.431 (6)
C9—C13	1.393 (5)	S21—C25	1.810 (5)
C9—C11	1.404 (5)	C25—F28A	1.274 (7)
C9—C15	1.484 (5)	C25—F27A	1.286 (7)
N10—C14	1.344 (5)	C25—F26A	1.296 (8)
N10—C12	1.356 (5)	C25—F28B	1.305 (10)
N10—H10	0.83 (4)	C25—F26B	1.322 (10)
C11—C12	1.409 (5)	C25—F27B	1.366 (11)
			
C2—C1—C11	121.1 (4)	C19—C18—C17	120.6 (5)
C2—C1—H1	119.4	C19—C18—H18	119.7
C11—C1—H1	119.4	C17—C18—H18	119.7
C1—C2—C3	121.2 (4)	C18—C19—C20	119.6 (5)
C1—C2—H2	119.4	C18—C19—H19	120.2
C3—C2—H2	119.4	C20—C19—H19	120.2
C4—C3—C2	120.7 (4)	C15—C20—C19	120.7 (5)
C4—C3—H3	119.7	C15—C20—H20	119.6
C2—C3—H3	119.7	C19—C20—H20	119.6
C3—C4—C12	119.1 (4)	O24B—S21—O23A	140.3 (5)
C3—C4—H4	120.5	O24B—S21—O24A	25.3 (4)
C12—C4—H4	120.5	O23A—S21—O24A	115.0 (3)
C6—C5—C14	118.3 (4)	O24B—S21—O22A	96.0 (5)
C6—C5—H5	120.9	O23A—S21—O22A	114.2 (2)
C14—C5—H5	120.9	O24A—S21—O22A	114.2 (4)
C5—C6—C7	122.1 (4)	O24B—S21—O22B	117.7 (4)
C5—C6—H6	119.0	O23A—S21—O22B	89.6 (4)
C7—C6—H6	119.0	O24A—S21—O22B	129.9 (4)
C8—C7—C6	120.6 (4)	O22A—S21—O22B	24.7 (4)
C8—C7—H7	119.7	O24B—S21—O23B	116.8 (2)
C6—C7—H7	119.7	O23A—S21—O23B	24.6 (4)
C7—C8—C13	119.7 (4)	O24A—S21—O23B	91.9 (4)
C7—C8—H8	120.1	O22A—S21—O23B	136.2 (4)
C13—C8—H8	120.1	O22B—S21—O23B	112.0 (2)
C13—C9—C11	119.4 (3)	O24B—S21—C25	97.5 (5)
C13—C9—C15	121.0 (3)	O23A—S21—C25	101.4 (5)
C11—C9—C15	119.6 (3)	O24A—S21—C25	109.7 (4)
C14—N10—C12	124.4 (3)	O22A—S21—C25	100.2 (4)
C14—N10—H10	118 (3)	O22B—S21—C25	106.7 (3)
C12—N10—H10	117 (3)	O23B—S21—C25	103.1 (4)
C9—C11—C12	119.8 (4)	F28A—C25—F27A	108.7 (5)
C9—C11—C1	123.7 (3)	F28A—C25—F26A	108.1 (5)
C12—C11—C1	116.5 (3)	F27A—C25—F26A	107.4 (5)
N10—C12—C11	118.2 (4)	F28A—C25—F28B	25.3 (4)
N10—C12—C4	120.4 (4)	F27A—C25—F28B	86.5 (6)
C11—C12—C4	121.4 (4)	F26A—C25—F28B	128.2 (8)
C9—C13—C14	119.7 (3)	F28A—C25—F26B	85.4 (5)
C9—C13—C8	122.7 (4)	F27A—C25—F26B	126.3 (8)
C14—C13—C8	117.5 (4)	F26A—C25—F26B	25.0 (4)
N10—C14—C5	119.8 (4)	F28B—C25—F26B	108.6 (6)
N10—C14—C13	118.4 (3)	F28A—C25—F27B	123.3 (9)
C5—C14—C13	121.7 (4)	F27A—C25—F27B	24.4 (4)
C20—C15—C16	118.3 (4)	F26A—C25—F27B	83.7 (5)
C20—C15—C9	121.3 (4)	F28B—C25—F27B	105.9 (7)
C16—C15—C9	120.3 (4)	F26B—C25—F27B	105.0 (6)
C15—C16—C17	120.5 (5)	F28A—C25—S21	114.3 (5)
C15—C16—H16	119.8	F27A—C25—S21	108.4 (5)
C17—C16—H16	119.8	F26A—C25—S21	109.7 (6)
C18—C17—C16	120.2 (5)	F28B—C25—S21	112.4 (7)
C18—C17—H17	119.9	F26B—C25—S21	111.8 (6)
C16—C17—H17	119.9	F27B—C25—S21	112.6 (9)
			
C11—C1—C2—C3	−0.3 (7)	C16—C17—C18—C19	−0.2 (9)
C1—C2—C3—C4	−0.1 (7)	C17—C18—C19—C20	0.1 (8)
C2—C3—C4—C12	0.3 (7)	C16—C15—C20—C19	0.4 (7)
C14—C5—C6—C7	1.8 (7)	C9—C15—C20—C19	177.4 (4)
C5—C6—C7—C8	−0.1 (7)	C18—C19—C20—C15	−0.2 (8)
C6—C7—C8—C13	−0.8 (7)	O24B—S21—C25—F28A	83.8 (5)
C13—C9—C11—C12	1.2 (5)	O23A—S21—C25—F28A	−61.2 (5)
C15—C9—C11—C12	−177.6 (3)	O24A—S21—C25—F28A	60.8 (6)
C13—C9—C11—C1	−177.2 (4)	O22A—S21—C25—F28A	−178.7 (5)
C15—C9—C11—C1	4.0 (6)	O22B—S21—C25—F28A	−154.2 (5)
C2—C1—C11—C9	178.8 (4)	O23B—S21—C25—F28A	−36.0 (5)
C2—C1—C11—C12	0.4 (6)	O24B—S21—C25—F27A	−37.7 (6)
C14—N10—C12—C11	−3.7 (6)	O23A—S21—C25—F27A	177.4 (5)
C14—N10—C12—C4	176.5 (4)	O24A—S21—C25—F27A	−60.6 (6)
C9—C11—C12—N10	1.6 (5)	O22A—S21—C25—F27A	59.9 (6)
C1—C11—C12—N10	−180.0 (4)	O22B—S21—C25—F27A	84.3 (5)
C9—C11—C12—C4	−178.6 (4)	O23B—S21—C25—F27A	−157.5 (5)
C1—C11—C12—C4	−0.1 (6)	O24B—S21—C25—F26A	−154.7 (6)
C3—C4—C12—N10	179.6 (4)	O23A—S21—C25—F26A	60.4 (6)
C3—C4—C12—C11	−0.2 (6)	O24A—S21—C25—F26A	−177.6 (6)
C11—C9—C13—C14	−2.0 (5)	O22A—S21—C25—F26A	−57.1 (6)
C15—C9—C13—C14	176.7 (3)	O22B—S21—C25—F26A	−32.7 (6)
C11—C9—C13—C8	176.7 (4)	O23B—S21—C25—F26A	85.5 (6)
C15—C9—C13—C8	−4.5 (6)	O24B—S21—C25—F28B	56.2 (5)
C7—C8—C13—C9	−178.7 (4)	O23A—S21—C25—F28B	−88.7 (6)
C7—C8—C13—C14	0.0 (6)	O24A—S21—C25—F28B	33.3 (6)
C12—N10—C14—C5	−177.6 (4)	O22A—S21—C25—F28B	153.7 (6)
C12—N10—C14—C13	2.8 (6)	O22B—S21—C25—F28B	178.2 (5)
C6—C5—C14—N10	177.8 (4)	O23B—S21—C25—F28B	−63.6 (5)
C6—C5—C14—C13	−2.7 (6)	O24B—S21—C25—F26B	178.7 (5)
C9—C13—C14—N10	0.1 (5)	O23A—S21—C25—F26B	33.7 (6)
C8—C13—C14—N10	−178.7 (4)	O24A—S21—C25—F26B	155.8 (5)
C9—C13—C14—C5	−179.5 (4)	O22A—S21—C25—F26B	−83.8 (6)
C8—C13—C14—C5	1.8 (5)	O22B—S21—C25—F26B	−59.3 (5)
C13—C9—C15—C20	82.2 (5)	O23B—S21—C25—F26B	58.9 (5)
C11—C9—C15—C20	−99.1 (5)	O24B—S21—C25—F27B	−63.4 (6)
C13—C9—C15—C16	−100.9 (5)	O23A—S21—C25—F27B	151.7 (5)
C11—C9—C15—C16	77.9 (5)	O24A—S21—C25—F27B	−86.3 (6)
C20—C15—C16—C17	−0.6 (7)	O22A—S21—C25—F27B	34.1 (6)
C9—C15—C16—C17	−177.6 (4)	O22B—S21—C25—F27B	58.6 (5)
C15—C16—C17—C18	0.5 (8)	O23B—S21—C25—F27B	176.8 (5)

### Hydrogen-bond geometry (Å, °)

**Table:**

Cg2 is the centroid of the C1–C4/C11/C12 ring.

**Table:**

*D*—H···*A*	*D*—H	H···*A*	*D*···*A*	*D*—H···*A*
C3—H3···O24A^i^	0.93	2.44	3.333 (9)	160
C4—H4···O22A	0.93	2.59	3.348 (8)	139
C5—H5···O23A	0.93	2.28	3.154 (9)	157
N10—H10···O22A	0.83 (4)	2.43 (4)	3.198 (9)	154 (3)
C17—H17···*Cg*2^ii^	0.93	2.99	3.632 (7)	127
C20—H20···O24A^iii^	0.93	2.56	3.461 (9)	162

Symmetry codes:  (i) −*x*+1, −*y*+2, −*z*+1; (ii) −*x*+3/2, *y*−1/2, −*z*+1/2; (iii) −*x*+1, −*y*+1, −*z*+1.

### Table 2 C–F···π and S–O···π interactions (Å, °).

Cg1 and Cg3 are the centroids of the C9/N10/C11–C14 and C5–C8/C13/C14 rings, respectively.

**Table:**

*X*	*I*	*J*	*I*···*J*	*X*···*J*	*X*–*I*···*J*
C25	F26A	*Cg*3^iv^	3.855 (11)	3.987 (6)	86.3 (5)
C25	F28A	*Cg*3^iv^	3.501 (6)	3.987 (6)	103.1 (4)
S21	O22A	*Cg*3^iii^	3.617 (9)	3.990 (2)	94.7 (4)
S21	O23A	*Cg*1^iii^	3.125 (11)	3.923 (2)	114.7 (5)
C25	F26B	*Cg*3^iv^	3.743 (13)	3.987 (6)	90.8 (6)
C25	F28B	*Cg*3^iii^	3.545 (13)	3.987 (6)	100.2 (6)
S21	O22B	*Cg*3^iii^	3.387 (7)	3.990 (2)	104.7 (3)
S21	O23B	*Cg*1^iii^	3.159 (9)	3.923 (2)	111.8 (4)

Symmetry codes: (iii) –*x* + 1, –*y* + 1, –*z* + 1; (iv) –*x* + 2, –*y* + 1, –*z* + 1.
